# splitGFP Technology Reveals Dose-Dependent ER-Mitochondria Interface Modulation by α-Synuclein A53T and A30P Mutants

**DOI:** 10.3390/cells8091072

**Published:** 2019-09-12

**Authors:** Tito Calì, Denis Ottolini, Mattia Vicario, Cristina Catoni, Francesca Vallese, Domenico Cieri, Lucia Barazzuol, Marisa Brini

**Affiliations:** 1 Department of Biomedical Sciences, University of Padova, Padova 35131, Italy; mattiavicario@hotmail.it (M.V.); francesca.vallese84@gmail.com (F.V.); domenico.cieri87@gmail.com (D.C.); luciabarazzuol@gmail.com (L.B.); 2 Padova Neuroscience Center (PNC), University of Padova, Padova 35131, Italy; 3 Department of Biology, University of Padova, Padova 35131, Italy; ottolinidenis@gmail.com (D.O.); cristina.catoni@phd.unipd.it (C.C.)

**Keywords:** Parkinson’s disease, alpha-synuclein, calcium, mitochondria, ER-mitochondria contact sites

## Abstract

Familial Parkinson’s disease (PD) is associated with duplication or mutations of α-synuclein gene, whose product is a presynaptic cytosolic protein also found in mitochondria and in mitochondrial-associated ER membranes. We have originally shown the role of α-syn as a modulator of the ER-mitochondria interface and mitochondrial Ca^2+^ transients, suggesting that, at mild levels of expression, α-syn sustains cell metabolism. Here, we investigated the possibility that α-syn action on ER-mitochondria tethering could be compromised by the presence of PD-related mutations. The clarification of this aspect could contribute to elucidate key mechanisms underlying PD. The findings reported so far are not consistent, possibly because of the different methods used to evaluate ER-mitochondria connectivity. Here, the effects of the PD-related α-syn mutations A53T and A30P on ER-mitochondria relationship were investigated in respect to Ca^2+^ handling and mitochondrial function using a newly generated SPLICS sensor and aequorin-based Ca^2+^measurements. We provided evidence that A53T and A30P amino acid substitution does not affect the ability of α-syn to enhance ER/mitochondria tethering and mitochondrial Ca^2+^ transients, but that this action was lost as soon as a high amount of TAT-delivered A53T and A30P α-syn mutants caused the redistribution of α-syn from cytoplasm to foci. Our results suggest a loss of function mechanism and highlight a possible connection between α-syn and ER-mitochondria Ca^2+^ cross-talk impairment to the pathogenesis of PD.

## 1. Introduction

Structural alterations and toxic misfolding of susceptible proteins are common hallmarks of many neurodegenerative diseases, including amyotrophic lateral sclerosis, Alzheimer’s, Parkinson’s, and Huntington’s disease, and are linked to the degeneration and death of specific neuronal populations in the human brain [1,2]. Parkinson’s disease (PD) is characterized by loss of dopaminergic neurons of the substantia nigra pars compacta in ventral mid-brain [3] and accumulation of intra-cytoplasmic fibrillary aggregates, termed as Lewy bodies, mainly constituted by α-synuclein (α-syn) [4]. α-syn is a 140-amino acid unfolded protein highly expressed in the nervous system with a preferential distribution at the presynaptic terminals [5]. PD familial studies have identified a number of α-syn mutations, leading to either an early (A53T, A30P, E46K, G51D) or a late (H50Q) onset of the disease [6]. Besides its role in neuronal synaptic transmission [7,8,9] the function of α-syn within the cells is not yet fully understood. Under pathological conditions, monomeric cytosolic α-syn undergoes structural changes that cause its aggregation and insolubility [10,11,12,13,14,15] typically observed in many forms of neurodegeneration [16,17]. Several studies have shown that α-syn interacts with membrane phospholipids and that it can selectively bind to mitochondrial sub-compartments [18,19,20,21,22,23]. Mitochondrial dysfunctions are a common element in the pathogenesis of many neurodegenerative diseases, including PD [24], being pivotal mitochondrial processes directly influenced by α-syn [25]. Alterations in mitochondrial phenotypes have been consistently reported in mutant α-syn transgenic [26,27] and null mice [28], as well as in model cells overexpressing wt and mutant α-syn [29]. α-syn has also been shown to participate in the maintenance of mitochondrial integrity by regulating the fission/fusion machinery and the autophagic process [30,31,32,33]. Interestingly, we have previously demonstrated that α-syn positively enhanced mitochondrial Ca^2+^ transients generated upon Ca^2+^ release from the endoplasmic reticulum (ER) by increasing the ER-mitochondria contact sites in a dose-dependent manner [34]. A dose-dependent effect has been confirmed also for α-syn modulation of other mitochondria-related activities [22,35,36]. Although prevalently cytosolic, α-syn is present in the nucleus [37,38,39], in the mitochondria [19,20,40,41] and in the mitochondria-associated ER membranes (MAMs) fraction [30,42]. Despite its ability to affect ER-mitochondria tethering, a general consensus on the precise action of α-syn at this interface is still lacking and it is unclear whether it interferes directly or indirectly with the tethering machinery. The possibility that a loss of function mechanism could be in place, thus explaining the different findings [30,34,42], is therefore interesting. In the present study, in order to provide further insights into the role of α-syn in key mechanisms underlying PD, we investigated the effects of A53T and A30P α-syn PD-related mutants on ER-mitochondria associations and mitochondrial Ca^2+^ handling. We have found that the aggregation-prone A53T and A30P mutants recapitulate the previously observed effect of WT α-syn [34]. Interestingly, the A53T and A30P α-syn mutants (reported to be more susceptible to aggregation) were able to increase the number of ER-mitochondria contact sites (as clearly documented by our newly generated split-GFP based ER-mitochondria contact sensor (SPLICS)) [43] and enhance mitochondria Ca^2+^ transients in our cell model under conditions in which their distribution is cytosolic. However, their ability to positively modulate mitochondrial Ca^2+^ transients was lost when their redistribution occurred. These results indicate that the increased aggregation propensity of the α-syn mutants is a key element in the pathogenesis of PD since it might lead to premature sequestration of α-syn into non-functional aggregates and through a loss-of-function mechanism affect the ER-mitochondria interface and, in turn, essential mitochondrial functions.

## 2. Materials and Methods

### 2.1. DNA Constructs

Plasmids encoding wt and mutant α-syn and TAT-fusion wt and mutant α-syn recombinant proteins were previously described [34,44]. Briefly, the SPLICS-P2A construct has been generated by cloning the ER_short_-β_11_ and the OMM-GFP_1–10_ coding sequences described in [43], respectively, upstream and downstream of a viral 2A peptide sequence contained in the pSYC-181 plasmid (Addgene, Watertown, MA, USA), previously reported to be cleaved within the cell to generate an equimolar amount of the two genes [45]. From this construct, named pSYC-SPLICS_S_-P2A [43], the sequence encoding SPLICS-P2A was excised using BamHI and XbaI restriction enzymes and subcloned in mammalian expression vector pCDNA3.1. All the constructs were verified by sequencing. Mitochondria-targeted GFP (mtGFP) expression vector was kindly provided by Prof. R. Rizzuto, University of Padova [46]. Plasmids encoding recombinant targeted aequorin probes were previously described, cytAEQ in [47], mtAEQ in [48] and erAEQ in [49]. For a comprehensive view of the tools and the methodology please refer to [50,51,52,53].

### 2.2. Cell lines and Transfection

HeLa cells were maintained in DMEM (Euroclone, Milan, Italy) supplemented with 10% FBS (Euroclone, Milan), 100 units/mL penicillin, and 100 μg/mL streptomycin, and kept at 37 °C in a humidified atmosphere of 5% CO_2_. Cells were seeded onto 13-mm (for aequorin measurements) or 24-mm (for ER-mitochondria contact sites analysis) glass coverslips for 12 h before transfection. For [Ca^2+^] measurements, HeLa cells were co-transfected by calcium-phosphate procedure with aequorin encoding plasmids and pcDNA3 empty plasmid (mock) or α-syn expressing vectors in a 1:2 ratio as previously described [34]. Ca^2+^ measurements were performed 36 h later.

Cells plated for Western blotting were collected 24–36 h after transfection. For TAT-mediated delivery, recombinant TAT fusion proteins were added directly onto the seeded aequorin-transfected cells and incubated for 2.5–5 h in DMEM, 10% FBS, and antibiotics at 37 °C in a 5% CO_2_ atmosphere. After incubation with TAT fusion proteins, the cells were extensively washed with PBS before starting Ca^2+^ measurements [34].

### 2.3. Western Blotting 

HeLa cells were flooded on ice with 20 mM ice-cold *N*-ethylmaleimide in PBS to prevent post-lysis oxidation of free cysteines. Cell extracts were prepared by solubilizing cells in ice-cold 2% CHAPS in Hepes-buffered saline (50 mM HEPES, 0.2 M NaCl, pH 6.8) containing *N*-ethylmaleimide, 1 mM PMSF, and mixture protease inhibitors (Sigma, St. Louis, MO, USA). Postnuclear supernatants were collected after centrifugation 10 min at 10,000× *g* at 4 °C. The total protein content was determined by the Bradford assay (Bio-Rad, Hercules, CA, USA). Samples were loaded on a 15% SDS-PAGE Tris/HCl gel, transferred onto PVDF membranes (Bio-Rad, Hercules, CA, USA), and incubated overnight with the specific primary antibody at 4 °C. Detection was carried out by incubation with secondary horseradish peroxidase-conjugated anti-rabbit or anti-mouse IgG antibody (Santa Cruz Biotechnology, Dallas, TX, USA) for 1.5 h at room temperature. The proteins were visualized by the chemiluminescent reagent Immobilon Western (Merck KGaA, Darmstadt, Germany). Mouse monoclonal anti-α-syn antibody (sc-12767, Santa Cruz Biotechnology, Inc.) was used at a 1:30 dilution in immunocytochemistry analysis and at a 1:500 dilution in Western blotting analysis. Mouse monoclonal anti-β-actin (AC-15, Merck KGaA, Darmstadt, Germany) was used at a 1:90.000 dilution in Western blotting. 

### 2.4. Immunocytochemistry Analysis

Transfected or TAT α-syn loaded HeLa cells plated on coverslips were fixed with 3.7% formaldehyde in phosphate-buffered saline (PBS; 140 mM NaCl, 2 mM KCl, 1.5 mM KH_2_PO_4_, 8 mM Na_2_HPO_4_, pH 7.4) for 20 min and washed three times with PBS. Cell permeabilization was performed by 20 min of incubation in 0.1% Triton X-100 PBS followed by 30 min wash in 1% gelatin (type IV, from bovine skin, Merck KGaA, Darmstadt, Germany) in PBS at room temperature. The coverslips were then incubated for 90 min at 37 °C in a wet chamber with the specific antibody diluted in PBS. Staining was revealed by the incubation with specific AlexaFluor 488 or 594 secondary antibodies for 45 min at room temperature (1:100 dilution in PBS; Thermo Fisher Scientific, Waltham, MA, USA). Fluorescence was analyzed with a Zeiss Axiovert microscope equipped with a 12-bit digital cooled camera (Micromax-1300Y; Princeton Instruments Inc., Trenton, NJ, USA) or Leica Confocal SP5 microscope. Images were acquired by using Axiovision 3.1 or Leica AS software (Leica Microsystems, Wetzlar, Germany).

### 2.5. Aequorin Measurements

Mitochondrial low-affinity aequorin (mtAEQ) and cytosolic wt aequorin (cytAEQ) were reconstituted by incubating cells for 3 h (cytAEQ) or 1.5 h (mtAEQ) with 5 μM wt coelenterazine (Invitrogen) in DMEM supplemented with 1% fetal bovine serum at 37 °C in a 5% CO_2_ atmosphere. To functionally reconstitute low-affinity ER-targeted aequorin (erAEQ), the ER Ca^2+^ content had to be drastically reduced. To this end, cells were incubated for 1.5 h at 4 °C in Krebs–Ringer modified buffer (KRB, 125 mM NaCl, 5 mM KCl, 1 mM Na_3_PO_4_, 1 mM MgSO_4_, 5.5 mM glucose, 20 mM HEPES, pH 7.4, 37 °C) supplemented with the Ca^2+^ ionophore ionomycin (5 μM), 600 μM EGTA, and 5 μM coelenterazine (Thermo Fisher Scientific, Waltham, MA, USA). Cells were then extensively washed with KRB supplemented with 2% bovine serum albumin and 1 mM EGTA [53]. After reconstitution, cells were transferred to the chamber of a purpose-built luminometer, and Ca^2+^ measurements were started in KRB medium added with 1 mMCaCl_2_ or 100 μM EGTA or 1 mM EGTA according to the different protocols and aequorin probes. 100 μM histamine was added, as specified in the figure legends. All the experiments were terminated by cell lysis with 100 μM digitonin in a hypotonic Ca^2+^-rich solution (10 mM CaCl_2_ in H_2_O) to discharge the remaining reconstituted active aequorin pool. The light signal was collected and calibrated off-line into Ca^2+^concentration values, as previously described [47,54].

### 2.6. ER-Mitochondria Contact Site Analysis

Cells plated on 13-mm-diameter coverslips were transfected with SPLICS [43] together with empty or WT or mutants α-syn expressing vectors or incubated with TAT α-syn upon the transfection with SPLICS. Fluorescence was analyzed 48–72 h after transfection with a Leica TSC SP5 inverted confocal microscope, using HCX PL APO 63X/numerical aperture 1.40–0.60 upon excitation at 488 nm. Images were acquired by using the Leica AS software. To count ER–mitochondria contacts, a complete z-stack of the cell was acquired every 0.29 µm. Z-stacks were processed using Fiji [55]. Images were first convolved, and then filtered using the Gaussian blur filter. A 3D reconstruction of the resulting image was obtained using the Volume J plugin (http://bij.isi.uu.nl/vr.htm). A selected face of the 3D rendering was then thresholded and used to count ER–mitochondria contact sites as already described [43,56].

### 2.7. Statistical Analysis

Data are given as means ± SD (standard deviation). Where multiple groups were compared, statistical significance was calculated by one-way ANOVA with a post hoc Dunett correction. Normally distributed data were analyzed using ANOVA and unpaired Student’s two-tailed *t*-test for two-group comparison with no correction assuming the same SD. All statistical significance was calculated at *p* = 0.05, using GraphPad Prism 6 (Graphpad, San Diego, CA, USA). For all the analysis, the samples were collected and processed simultaneously and, therefore, no randomization was appropriate. n = number of independent experiments or cells from at least three different transfection/treatments. When significant, *p*-values were stated in the figure legends. (GraphPad Prism, *** *p* < 0.0005, ** *p* < 0.001, and * *p* < 0.05). 

## 3. Results

### 3.1. α-syn A53T and A30P Mutants Physically Modulate ER-Mitochondria Contact Sites

**Figure 1:**
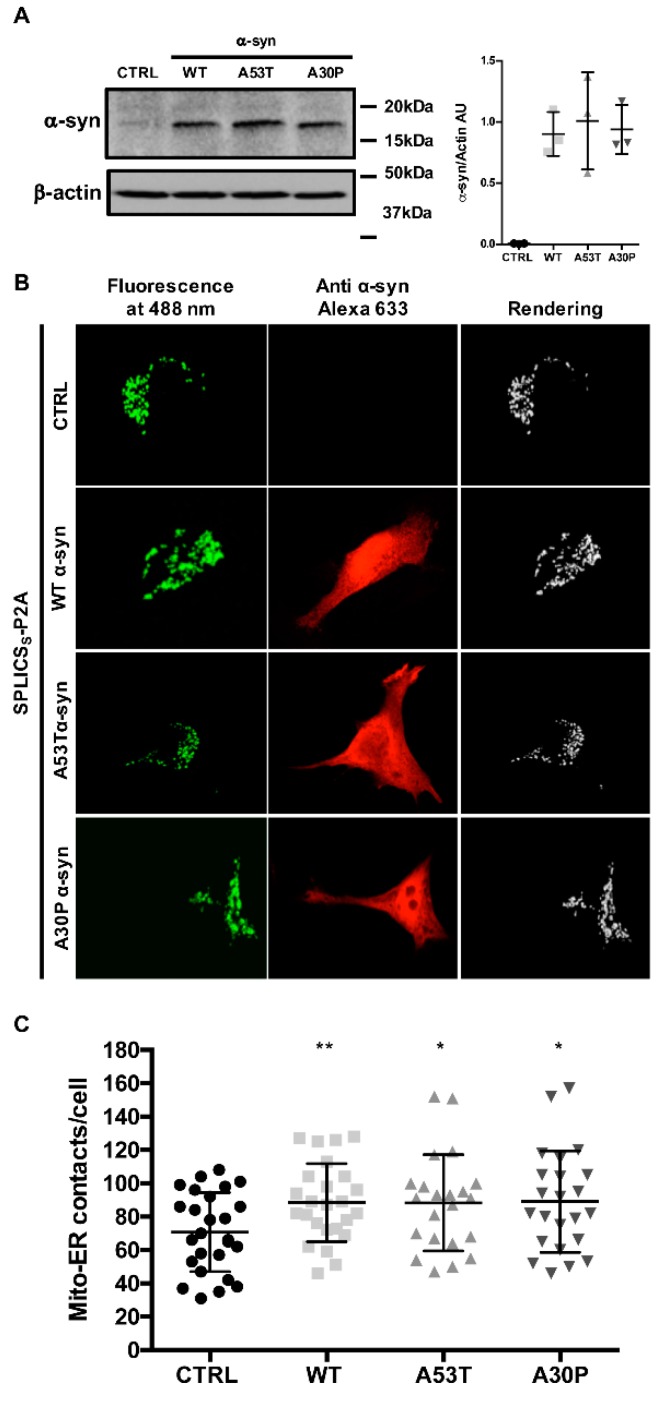
α-syn A53T and A30P mutants physically modulate ER-mitochondria contact sites. (**A**) HeLa cells were transfected with wt, A53T, and A30P α-syn expression plasmids and analyzed by Western blotting with an anti α-syn antibody. Equal loading was verified by probing the membrane with an anti β-actin antibody. Quantification of three independent experiments (**B**) HeLa cells were co-transfected with wt, A53T and A30P α-syn expression vectors and the SPLICS_S_ sensor to assess short-range ER-mitochondria associations. Reconstitution of the fluorescent signal was observed upon 488 nm wavelength excitation in α-syn positive cells probed with an anti α-syn primary antibody and revealed by an Alexa 633 secondary antibody. The 3D rendering of the Z-stacks acquired for the SPLICS_S_ probe is shown on the right. (**C**) Quantification of the ER-mitochondria contact sites/cell in the different conditions is shown as mean ± SD. *, *p* < 0.05, **, *p* < 0.01. One-way ANOVA test retrieved a *p*-value of 0.04. Unpaired Student’s two-tailed *t*-test was used for two-group comparison. No correction was applied since the same SD was assumed. Dunnet’s post-test was also applied to compare wt and α-syn mutants each other but no significance was detected. The asterisks refer to Student’s two-tailed *t*-test where the comparison was for each independent sample vs. mock cells.

We had previously demonstrated that mild α-syn overexpression promotes ER-mitochondria contact sites formation/stabilization and favors Ca^2+^ transfer from the ER to mitochondria, while its silencing causes mitochondrial impairments by loosening the ER-mitochondria interface [34]. Later on, other reports established that α-syn is also present at the MAMs and that PD-related mutations might contribute to the onset of the pathogenic phenotype by differentially interfering with MAMs functions [31,42]. Since we have hypothesized that exaggerated α-syn expression leads to loss of function at the ER-mitochondria interface, we decided to explore whether the familial PD-related A30P and A53T α-syn mutants could have an impact on the ER-mitochondria contact sites. We took advantage from the use of a novel splitGFP-based sensor for organelles proximity, the SPLICS, recently developed by our group [43] and based on the ability of two organelle targeted split-GFP fragments to reconstitute the GFP fluorescence when the membranes come in close proximity. As a result, a dotty pattern of fluorescence where organelle tethering occurs will be detected and quantified on 3D reconstructions of complete Z-stacks analysis. We have decided to test it in cells overexpressing A30P and A53T α-syn mutants, but at the same time, we have also repeated the experiments in HeLa cells overexpressing WT α-syn to confirm our previous data obtained by calculating Manders’ coefficient of mitochondrial-targeted RFP and ER targeted GFP [34]. First, the expression level and the sub-cellular localization of the overexpressed A53T and A30P α-syn mutants were analysed in HeLa cells at 36 h after transfection and compared to those detected in empty-vector- and in WT α-syn-transfected cells. As shown and quantified in Figure 1A, the endogenous expression level of α-syn in empty vector-transfected cells (ctrl) was barely detectable compared to that of WT, A53T, and A30P in α-syn-overexpressing HeLa cells. It is also appreciable that, in our experimental conditions, WT, A53T, and A30P α-syn mutants-overexpressing HeLa cells displayed either comparable protein expression levels (Figure 1A and quantification, values are: 0.0051 ± 0.0025 for control cells, 0.9021 ± 0.1797 for WT α-syn; 1.009 ± 0.3979 for A53T α-syn and 0.9390 ± 0.2006 for A53T α-syn, n = 3) and prevalent cytosolic distribution (Figure 1B), as determined by Western blotting and immunocytochemistry analysis, respectively. Equal loading of proteins was verified by probing the membrane with an anti-β-actin antibody. 

These findings allowed us to exclude that differences observed in the following experiments could be dependent on α-syn expression levels. Then, we monitored ER-mitochondria interactions occurring at a short-range distance (8–10 nm), i.e., those that are involved in ER-mitochondria Ca^2+^ transfer [57,58] in the presence of either WT, A30P, and A53T α-syn. As shown in Figure 1B (and quantified in Figure 1C), the expression of WT, A30P, and A53T α-syn, significantly increased the number of the tight ER-mitochondria interactions (number of ER-mitochondria contacts/cell: 70.6 ± 23.73 n = 26 for control cells; 88.4 ± 23.39 n = 25 for WT α-syn, *p* < 0.01; 88.3 ± 28.89 n = 22 for A53T α-syn *p* < 0.05; 89 ± 30.40 n = 23 for A30P α-syn *p* < 0.05). These results confirm our previous report on the positive effect of WT α-syn on the ER-mitochondria interface and demonstrate that the PD-related A30P and A53T amino acids substitutions do not affect the ability of α-syn to increase ER-mitochondria associations.

### 3.2. Overexpression of A53T and A30P α-Synuclein Mutants Enhances Mitochondrial Ca^2+^ Transients with the Same Extent than wt α-Synuclein

As previously documented by our group, the increase in ER-mitochondria tethering induced by WT α-syn overexpression was paralleled by increased mitochondria Ca^2+^ uptake upon cell stimulation with an InsP_3_-linked agonist [34]. We thus analyzed mitochondrial Ca^2+^ transients generated upon stimulation with 100 μM histamine, as well as the general Ca^2+^ handling of the cell in the presence of A53T and A30P α-syn. We performed Ca^2+^ measurements using organelle-targeted aequorin probes specific for the mitochondrial matrix (mtAEQ) (A), the cytoplasm (cytAEQ) (B) or the ER lumen (erAEQ) (C) [53].

**Figure 2:**
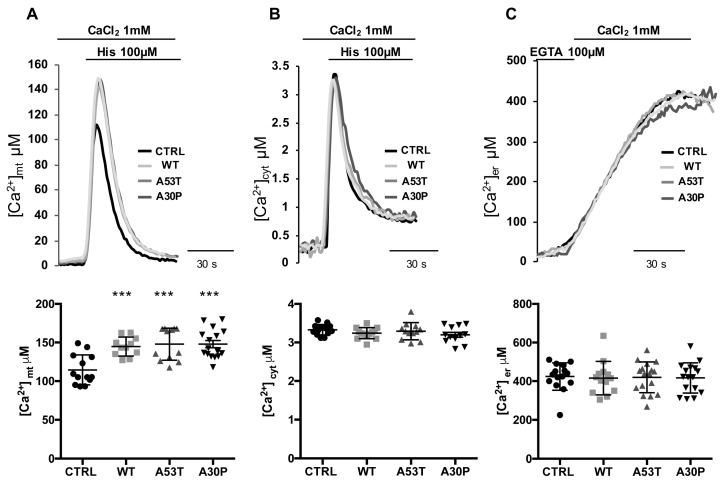
Overexpression of A53T and A30P α-synuclein mutants enhances mitochondrial Ca^2+^ transients. (**A**) Mitochondrial [Ca^2+^]_mt_, (**B**) cytosolic [Ca^2+^]_c_ Ca^2+^ transients, and (**C**) the kinetics of ER refilling upon re-addition of CaCl_2_ 1 mM to Ca^2+^-depleted cells (see Materials and Methods) in HeLa cells either mock-transfected or overexpressing A53T or A30P α-syn are shown. Cells were transfected with AEQ (either mitochondrial, cytosolic or targeted to the ER) (controls, mtAEQ, cytAEQ or erAEQ, respectively) or co-transfected with AEQ and WT or A53T or A30P α-syn. Traces refer to representative experiments selected from at least three independent experiments. Quantification of [Ca^2+^]_mt_, [Ca^2+^]_c_ and [Ca^2+^]_er_ in HeLa cells mock-transfected or overexpressing WT or A53T or A30P α-syn is shown at the bottom. Scatter plots represent the mean [Ca^2+^] peaks upon stimulation or after ER-refilling ± SD ***, *p* < 0.0005. One-way ANOVA test retrieved a *p*-value of 0.0001, for multiparametric analysis a Dunnet’s post-test was applied.

The overexpression of either A53T or A30P α-syn mutants resulted in significantly increased mitochondrial Ca^2+^ transients compared to the control transfected HeLa cells when exposed to the InsP_3_-linked agonist histamine (100 µM; Figure 2A top). However, quantification of individual mitochondrial Ca^2+^ responses revealed equal amplitude between A53T and A30P α-syn mutants (Figure 2A, bottom) ([Ca^2+^]_mt_ μM: 114.49 ± 19.37 n = 13 for control cells; 144 ± 12.31 n = 11 for WT α-syn, *p* < 0.001 vs. control; 147.88 ± 20.75 n = 12 for A53T α-syn, *p* < 0.001 vs. control; 147.85 ± 18.76 n = 16 for A30P α-syn, *p* < 0.001 vs. control). For comparison, similar Ca^2+^ measurements were performed on HeLa cells overexpressing WT α-syn, and also in this case, we observed an increase in mitochondrial Ca^2+^ transients upon cells stimulation. No differences were instead detected among control untransfected cells, WT α-syn and A53T or A30P α-syn mutants, respectively, in cytosolic Ca^2+^ transients generated upon histamine stimulation (Figure 2B) or ER Ca^2+^ content (Figure 2C) ([Ca^2+^]_c_ μM: 3.33 ± 0.13 n = 14 for control cells; 3.24 ± 0.14 n = 13 for WT α-syn; 3.29 ± 0.22 n = 12 for A53T α-syn; 3.20 ± 0.22 n = 12 for A30P α-syn; [Ca^2+^]_ER_ μM: 424 ± 71.15 n = 15 for control cells; 415.9 ± 86.61 n = 13 for WT α-syn; 419.9 ± 79.84 n = 17 for A53T α-syn; 416.7 ± 78.30 n = 16 for A30P α-syn), reinforcing the hypothesis that, as previously shown for WT α-syn [34], also in the case of the PD-related A53T or A30P α-syn mutants the modulation of the ER-mitochondria interface exclusively enhances mitochondrial Ca^2+^ transients, leaving cytosolic and ER Ca^2+^ levels unaffected.

### 3.3. α-syn A53T and A30P Mutants Enhance ER-Mitochondria Ca^2+^ Transfer but Impair Mitochondrial Ca^2+^ Uptake from the Extracellular Milieu

**Figure 3:**
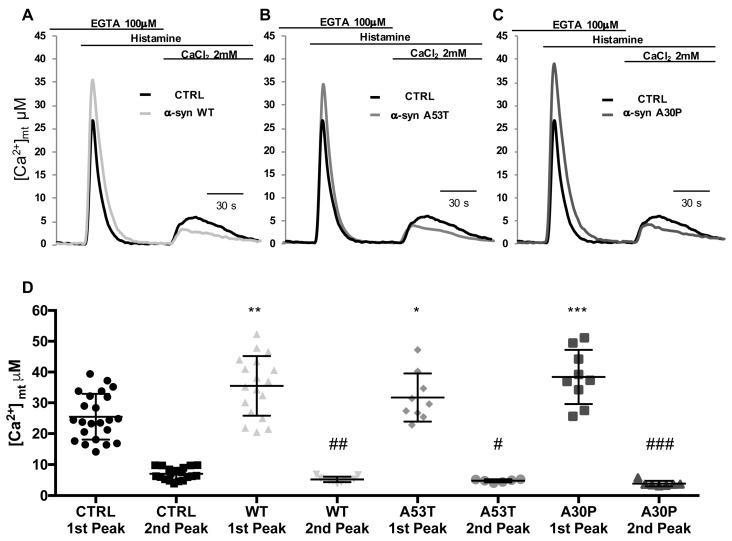
α-syn A53T and A30P mutants differently impinge on Ca^2+^ transients generated by ER Ca^2+^ release and Ca^2+^ influx from the extracellular milieu. HeLa cells were co-transfected with mtAEQ and WT α-syn (**A**) or A53T (**B**) or A30P (**C**) α-syn constructs or transfected with mtAEQ only (control). To discriminate the contribution of Ca^2+^ release from the ER and of Ca^2+^ influx from the extracellular ambient to the generation of [Ca^2+^]_mt_ transients, HeLa cells were perfused in KRB/EGTA 100 μM buffer and stimulated with histamine to release Ca^2+^ from the intracellular stores (first peak). Then, the perfusion medium was switched to KRB/CaCl_2_ 2 mM (in the continuous presence of histamine) to stimulate Ca^2+^ entry from the extracellular ambient (second peak). (**D**), Scatter plots represent mean [Ca^2+^] peak values upon stimulation ± SD. * and # *p* < 0.05, ** *p* < 0.01, ## *p* < 0.005 *** *p* < 0.0005, ###*p* < 0.001. The results are the mean of at least three independent experiments. One-way ANOVA test retrieved a *p*-value of 0.0001. Unpaired Student’s two-tailed *t*-test was used for two-group comparison. No correction was applied since the same SD was assumed. Dunnet’s post-test was also applied to compare wt and α-syn mutants each other but no significance was detected. 1^st^ and 2^nd^ peak mean values were analyzed separately. The asterisks refer to Student’s two-tailed *t*-test where the comparison was for each independent sample vs. mock cells.

The experiments shown above suggest that the mitochondrial Ca^2+^ uptake is indeed modulated by the overexpression of α-syn independently of PD-related A53T and A30P mutations. Nevertheless, the mitochondrial Ca^2+^ transients are shaped by the Ca^2+^ released from the ER and the Ca^2+^ entering from the extracellular milieu. To gain further insights into the specificity of the observed phenotype, these two contributions were analyzed separately. To this aim, HeLa cells overexpressing mitochondrial aequorin and either empty vector, WT α-syn or A53T or A30P mutants were perfused in KRB buffer containing 100 μM EGTA and stimulated with 100 μM histamine to generate a peak transient exclusively reflecting the mobilization of the ER Ca^2+^. Then the perfusion medium was switched to KRB supplemented with 2 mM CaCl_2_ (in the continuous presence of histamine), thus causing Ca^2+^ entry from the extracellular milieu that was primarily sensed by mitochondria located beneath the plasma membrane. Figure 3A–C for the representative traces of the experiments and D for the statistical analysis. The mitochondrial Ca^2+^ peaks in response to ER Ca^2+^ mobilization (first peak) was significantly higher in cells overexpressing in WT or A53T or A30P α-syn as compared with control cells, thus reinforcing the fact that α-syn-mediated mitochondrial Ca^2+^ modulation is dependent on its action in favoring the ER-mitochondria interface, ([Ca^2+^]_mt_ μM 1^st^ Peak: 25.46 ± 7.4 n = 23 for control cells; 35.47 ± 9.66 n = 18 for WT α-syn, *p* < 0.0006 vs. control; 31.7 ± 7.8 n = 9 for A53T α-syn, *p* < 0.05 vs. control; 38.39 ± 8.8 n = 9 for A30P α-syn, *p* < 0.001 vs. control (the significance was indicated with *)). The mitochondrial Ca^2+^ transients obtained in response to Ca^2+^ influx (second peak) were instead reduced in cells overexpressing WT or A53T or A30P α-syn (Figure 3A–C for representative traces of the experiments and D for the statistical analysis) [Ca^2+^]_mt_ μM 2^nd^ Peak: 6.99 ± 1.95 n = 19 for control cells; 5.19 ± 0.87 n = 11 for WT α-syn, *p* < 0.01 vs. control; 4.77 ± 0.48 n = 6 for A53T α-syn, *p* < 0.05 vs. control; 3.85 ± 0.86 n = 7 for A30T α-syn, *p* < 0.001 vs. control (the significance was indicated with #). The reduction in the peak generated by the re-addition of 2 mM CaCl_2_ is probably due to the α-syn-induced reduction of the influx pathways triggered by store depletion, as previously documented [59], and here, sensed mainly by mitochondria beneath the plasma membrane.

### 3.4. TAT-Mediated Delivery of A53T and A30P α-Synuclein Mutants Affects α-Syn Intracellular Distribution and Its Modulation of Mitochondrial Ca^2+^ Transients in a Dose-Dependent Manner

**Figure 4:**
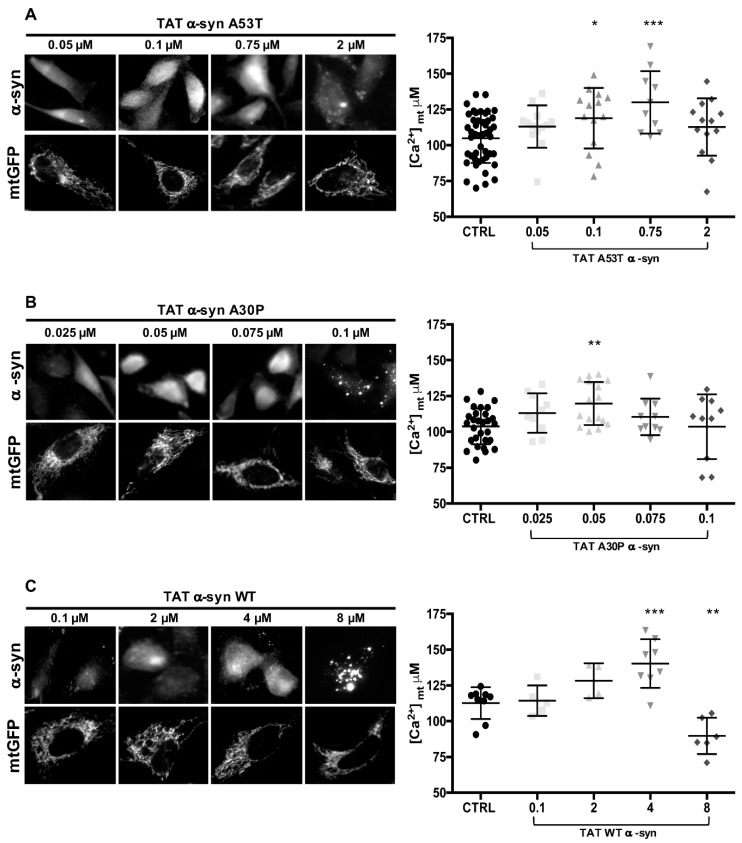
TAT-mediated delivery of A53T and A30P α-synuclein mutants induces dose-dependent α-syn redistribution and different effect on mitochondrial Ca^2+^ transients. HeLa cells were transfected with mtGFP or mtAEQ and then incubated with the indicated doses of TAT A53T (**A**) or TAT A30P (**B**) or TAT WT (**C**) α-syn. Immunolocalization of TAT mutant α-syn (**top**) and mitochondrial network (**bottom**) are revealed by α-syn primary antibodies and mtGFP fluorescence, respectively. The images revealing mitochondrial morphology were randomly acquired from TAT α-syn treated cells. Mitochondrial Ca^2+^ measurements were performed in HeLa cells upon treatment with TAT A53T or A30P or WT α-syn at the indicated doses. Panels on the right show the scatter plots representing the mean [Ca^2+^]_mt_ peak values upon cell stimulation with histamine. Results are the mean ± SD obtained from at least three independent experiments. *, *p* < 0.01; **, *p* < 0.005; ***, *p* < 0.0005. One-way ANOVA test retrieved a *p*-value of 0.0001, for multiparametric analysis a Dunnet’s post-test was applied.

Exogenous exposure of TAT WT α-syn fusion protein in HeLa cells was previously used to modulate intracellular α-syn content and shown to dose-dependently affect mitochondrial Ca^2+^ handling [34]. By following the same approach, we decided to use a TAT-mediated delivery system to fine-tune the intracellular levels of A53T and A30P α-syn mutants and to monitor mitochondrial Ca^2+^ transients in the same conditions. To this end, Hela cells were in parallel transfected with mtGFP or mtAEQ probes to specifically follow mitochondrial morphology and mitochondrial Ca^2+^ transients, respectively, and then incubated with TAT A53T or A30P at different doses (Figure 4). We selected ranges of TAT A53T (0.05 – 2 µM) and A30P (0.025 – 0.1 µM) concentrations at which we observed intracellular re-distribution of α-syn and monitored mitochondrial morphology by co-transfected mitochondrially targeted GFP (mtGFP in Figure 4A,B). 

None of the concentrations tested macroscopically affected mitochondrial morphology as shown by the mtGFP signal, suggesting that the α-syn levels were not strong enough to induce mitochondrial fission [32]. Immunocytochemistry analysis performed by incubating the cells with a primary antibody against α-syn revealed a dose-dependent increase of the diffuse cytosolic α-syn signal up to 0.75 µM TAT A53T (Figure 4A) and 0.075 µM TAT A30P (Figure 4B). Over these concentrations α-syn intracellular redistribution to localized cytoplasmic foci occurred (at 2 µM TAT A53T and 0.1 µM TAT A30P, respectively). Mitochondrial Ca^2+^ measurements in HeLa cells subjected to increasing doses of TAT A53T or A30P exhibited a significant rise in mitochondrial Ca^2+^ uptake at 0.1-0.75 µM TAT A53T (Figure 4A right) and 0.05 µM TAT A30P (Figure 4B right), i.e., when the α-syn distribution is still cytosolic, but as soon as cytoplasmic foci appears, mitochondrial Ca^2+^ transients amplitude decreases, suggesting that α-syn is not available anymore to support ER-mitochondria Ca^2+^ transfer. Notably, in the case of A30P mutant the reduction occurs already at 0.075 µM when cytosolic foci are not yet evident, possibly suggesting that the transition is occurring precisely at this point. ([Ca^2+^]_mt_ μM: 104.82 ± 17.13 n = 46 for control cells; 113.08 ± 14.77 n = 13 for 0.05 μM A53T α-syn; 118.89 ± 21.16 n = 14 for 0.1 μM A53T α-syn, *p* = 0.01; 130.04 ± 21.83 n = 10 for 0.75 μM A53T α-syn, *p* = 0.001; 112.81 ± 20.05 n = 13 for 2 μM A53T α-syn and 103.76 ± 12.49 n = 29 for control cells; 113.09 ± 13.79 n = 10 for 0.025μM A30P α-syn; 119.74 ± 15.02 n = 16 for 0.05 μM A30P α-syn, *p* < 0.005; 110.41 ± 12.80 n = 10 for 0.075 μM A30P α-syn; 103.6 ± 22.55 n = 10 for 0.1 μM A30P α-syn and 112.7 ± 11.15 n = 9 for control cells; 114.4 ± 10.64 n = 5 for 0.1 μM WT α-syn; 128.3 ± 12.16 n = 4 for 2 μM WT α-syn; 140.3 ± 17.01 n = 8 for 4 μM WT α-syn, *p* < 0.0005; 89.73 ± 12.72 n = 6 for 8 μM WT α-syn, *p* < 0.005). Similar results were found when titration of intracellular α-syn was obtained with TAT WT α-syn. For comparison, these data are shown in Figure 4C and their quantification on the right, in this case the appearance of cytoplasmic foci occurred upon incubation with 8 µM TAT WT.

**Figure 5:**
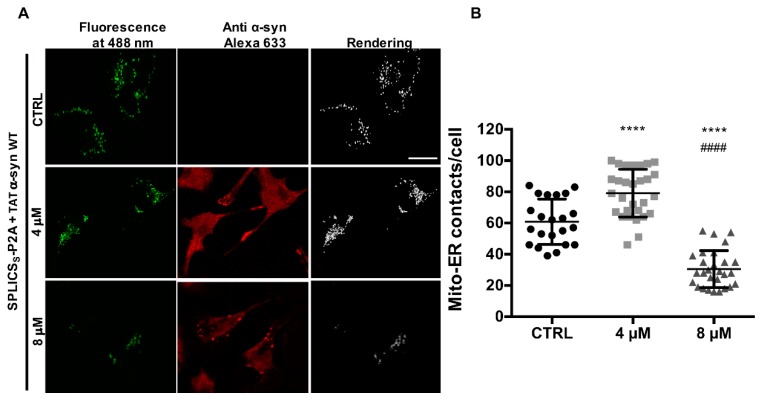
TAT-mediated delivery of α-syn affects ER-mitochondria contact sites in a dose-dependent manner. (**A**) HeLa cells were transfected with SPLICSs sensor to assess short-range ER-mitochondria associations and then incubated with the indicated doses of TAT WT α-syn. SPLICS_S_. Reconstitution of the fluorescent signal was observed upon 488 nm wavelength excitation in α-syn positive cells probed with an anti α-syn primary antibody and revealed by an Alexa 633 secondary antibody. The 3D rendering of the Z-stacks acquired for the SPLICS_S_ probe is shown on the right. (**B**) Quantification of the ER-mitochondria contact sites/cell in the different conditions is shown as mean ± SD. **** vs. CTRL or #### vs. 4 μM, *p* < 0.0001. One-way ANOVA test retrieved a *p*-value of 0.0001. Results are the mean ± SD obtained from at least two independent transfections, for multiparametric analysis a Dunnet’s post-test was applied.

As a proof of concept experiment, we decided to link the number of the ER-mitochondria contact sites with the α-syn aggregation state by exogenous exposure of TAT WT α-syn at the concentrations at which intracellular re-distribution of α-syn from cytosolic to intracellular foci was observed. As reported in Figure 5A and quantified in Figure 5B, the number of ER-mitochondria contact sites significantly increased upon expression of α-syn at concentrations in which a diffuse cytosolic signal was observed (i.e., at 4 μM TAT WT α-syn), in line with experiments of mitochondrial Ca^2+^ uptake shown in Figure 2A and Figure 4, thus enforcing the idea that increased levels of the protein can positively affect the number of ER-mitochondria contact sites. Interestingly enough, a drop was instead observed when redistribution to localized cytoplasmic foci occurred (i.e., at 8 μM TAT WT α-syn), suggesting that sequestration of functional α-syn into foci negatively affected the ER-mitochondria interface with a loss of function mechanism (number of ER-mitochondria contacts/cell: 60.86 ± 14.47 n = 22 for control cells; 79.14 ± 15.29 n = 29 for 4 μM TAT WT α-syn, *p* < 0.0001; 30.5 ± 11.82 n = 28 for 8 μM TAT WT α-syn *p* < 0.0001). Notably, redistribution of α-syn into foci induced a significant reduction of the ER-mitochondria contact sites as compared to control cells suggesting the intriguing possibility that also endogenous α-syn (or other important tethering factors as already suggested [60]) could indeed be redistributed into intracellular foci, thus affecting their physiological function. 

Altogether, these experiments indicate that the aggregation propensity of α-syn protein, through a loss of function mechanism impinging on the ER-mitochondria interface, may have a role in inducing mitochondrial Ca^2+^ signaling impairment that, in turn, could affect bioenergetic metabolism. 

## 4. Discussion

Parkinson’s disease (PD) affects six million individuals worldwide. The formation of intracellular inclusions of α-syn [61], whose autosomal dominant mutations [62] are found in familial forms of the disease, and the neuronal loss in the substantia nigra pars compacta [3] are the main hallmarks. Although prevalently cytosolic, α-syn is also present in different cellular locations such as the nucleus [37,38,39,63], the mitochondria [18,19,20,40,41,64] and the mitochondria-associated ER membranes (MAMs) fraction [30,42]. Its close relationship with mitochondria is also well established [25]. We have shown for the first time the involvement of α-syn in the modulation of mitochondrial Ca^2+^ handling and the ER-mitochondria communication [34]: It positively enhanced mitochondrial Ca^2+^ transients generated upon Ca^2+^ release from the endoplasmic reticulum (ER) by increasing the ER-mitochondria contact sites. Interestingly, we have proposed a dose-dependent mechanism of action that, more recently, has been confirmed to be in place for α-syn modulation of other mitochondrial functions [22,35,36]. To date, two additional studies have investigated the effect of α-syn on ER-mitochondria tethering directly [30,42], and they have reached different conclusions. The first study clearly showed that α-syn is indeed present in the MAM fraction and that its distribution in this location is altered by the PD-related mutations. Furthermore, by using confocal microscopy, they found that in M17 cells stably expressing the α-syn pathogenic mutants (A53T and A30P) the degree of ER-mitochondrial apposition was lower than in the cells transfected with the WT α-syn and the empty-vector control, suggesting that α-syn PD-related mutants may impinge on ER-mitochondria tethering by a gain of toxic function mechanism. Interestingly, when measured in HeLa cells, the ER-mitochondrial apposition was reduced also in the WT α-syn expressing cells compared to empty-vector control and to the same extent of the PD-related mutants [30], suggesting, on the other hand, that a loss of function mechanism could be involved instead (possibly related to the amount of overexpressed α-syn). The second study, by using EM, proximity ligation assays, and super resolution SIM methods, revealed that expression of WT and mutants (either A53T or A30P) α-syn decreased ER-mitochondria contacts to the same extent [42], again suggesting the occurrence of a loss of function mechanism. Although different appropriate methods have been used for quantifying contacts over the years, none of those mentioned above, nor among the existing ones, is still able to fulfill the essential requirements ensuring the best conditions to properly quantify the ER-mitochondria interactions: A good resolution coupled with the possibility to detect them in the most physiological condition, i.e., in living cells. This could certainly account for the discrepancies observed within the same or between different studies. Nevertheless, additional fundamental factors could be the key to understand the mechanism/s by which α-syn affect the ER-mitochondria interface, i.e., the aggregation tendency of α-syn itself and how the PD-related mutations affect this behavior. Indeed, none of the above-mentioned studies addresses the question following ER-mitochondria interaction both in the presence of different amounts of α-syn protein and monitoring its intracellular distribution to correlate its effects with changes in its aggregation propensity or with its soluble state [65,66,67]. In our previous study [34], we were able to fine-tune the intracellular levels of α-syn either by mild transient transfection conditions, i.e., the calcium phosphate procedure, or by artificially or pharmacologically increasing them. Under those conditions, we found a correlation between aggregation propensity of WT α-syn and its effects on mitochondrial Ca^2+^ uptake. Although indirectly, we hypothesized that this effect was dependent on the modulation of ER-mitochondria contact sites, evaluated by calculating Menders’ coefficient upon overexpression of two organelle targeted fluorescent proteins [34]. Here, we extended our previous analysis to the α-syn PD-related pathogenic mutations. To this aim, we employed the SPLICS probe that we have recently developed [43] and widely used in other studies by us and others to quantify the ER-mitochondria interactions under physiological conditions [43,56,68,69,70,71,72]. Our results confirmed the ability of WT α-syn to increase the ER-mitochondria contact sites and revealed that the introduction of A53T and A30P PD-related mutations does not abolish this action. In α-syn A53T and A30P expressing cells, the increase in ER-mitochondria interactions was also paralleled by a concomitant increase in mitochondria Ca^2+^ uptake upon cell stimulation with an InsP_3_ generating agonist which mobilized Ca^2+^ from the ER, suggesting that the PD-related mutants, when expressed at low levels, retain the ability to exert their effect at the MAMs. Noteworthy, this was also the main difference with the above-mentioned studies which employed Lipofectamine-based methods to induce high α-syn expression or selected highly expressing stable clones. To enforce our original idea that impairment in this ability could be due to a loss of function mechanism dependent on the sequestration of functional α-syn in aggregates rather than to the presence of mutations that could affect the ER-mitochondria tethering machinery, we induced α-syn intracellular re-distribution by exogenously applying an increasing amount of recombinant TAT WT, TAT A53T, and TAT A30P α-syn. Mitochondrial Ca^2+^ uptake upon cells stimulation was measured in these conditions. We had previously identified the TAT WT α-syn is able to positively modulate ER-mitochondria contact sites and mitochondrial Ca^2+^ uptake when applied at doses in the range of 4 µM. We had also previously shown that when TAT WT α-syn was applied at high doses, i.e., 8 µM, the diffuse cytosolic α-syn cellular distribution was compromised and the modulatory effect was lost, suggesting a loss of function mechanism [34]. Now, by applying an increasing amount of TAT A53T, and A30P α-syn we found the appearance of intracellular foci and/or aggregates of α-syn at lower concentrations, i.e., in the range of 0.1–2 µM, being the concentration even lower for the A30P in respect with the A53T mutant. These data indicate that these α-syn mutants are indeed characterized by an intrinsically higher aggregation/oligomerization propensity than WT α-syn, as already reported [65,66,73]. However, although many studies showed that A53T [66,74,75] aggregate more rapidly than WT α-synuclein in vitro using recombinant proteins and that more fibrils could be detected in cells [76,77], A30P [66,75] was found to aggregate to the same or a lesser extent than WT α-synuclein in vitro and form the same number of fibrils in cells. Thus, the question of their aggregation propensity in respect to the disease relevance needs to be further dissected. A recent study showed that the types of oligomers formed by A30P and A53T are different [78] and, interestingly, it has been also reported that the A30P, E46K, H50Q, G51D, and A53T mutants exhibited identical propensities to oligomerize in living cells, but had distinct abilities to form inclusions. While the A30P mutant reduced the percentage of cells with inclusions, the E46K mutant had the opposite effect [76], thus offering different experimental models to study oligomers/aggregates-induced cell toxicity. Noteworthy, in a proof-of-concept experiment, the number of ER-mitochondria contact sites was measured in conditions in which the amount of WT α-syn was fine-tuned to either increase its intracellular level without any apparent change in its cytosolic distribution or to promote strong subcellular re-localization into foci/aggregates. Under those conditions, we could observe a statistically significant increase (in the former) or a decrease (in the latter), respectively. These findings argue in favor of the hypothesis that α-syn could play a direct role in the modulation of the ER-mitochondria interface and suggest that protein aggregation could be responsible for clustering overexpressed, as well as endogenous α-syn (or important factors or tethering factors [60]), and compromising its action according to a loss of function mechanism. Interestingly, it should be noticed that an intracellular threshold of α-syn should also be reached in order to recapitulate the observed phenotypes. Indeed, despite the differences observed between the WT and the mutant forms of α-syn in the concentrations applied for the TAT-mediated delivery, upon transient transfection the cytosolic distribution of the protein (no matter whether WT or mutant) was sufficient to sustain increased ER-mitochondria contacts and mitochondrial Ca^2+^ transients. This suggests the intriguing possibility that the intracellular level of α-syn must indeed be tightly regulated and, although it is difficult to directly link the intracellular concentration of the protein with the phenotype, it could be argued that by transient transfection we are below the threshold required to induce the foci formation but α-syn levels are sufficient to positively modulate ER-mitochondria contacts and mitochondrial Ca^2+^. In this context it will be interesting to investigate whether the modulation of ER/mitochondria contact sites and mitochondrial Ca^2+^ handling could be differentially affected not only dose-dependently, as shown in the present paper, but also by the different α-syn species, i.e., oligomers rather than fibrils or amorphous aggregates. At the moment, these kinds of studies in a cell context are very difficult, however, the possibility that the α-syn-induced aggregates are not toxic per se and that impairments in the ER-mitochondria interface are early events preceding aggregation is fascinating and certainly deserves further investigations. 

## 5. Conclusions

These results suggest that the ability of α-syn to act as a positive regulator of the ER-mitochondria interface is not affected by the PD-related mutations A53T and A30P, rather, their increased aggregation propensity prevents α-syn from exerting its activity at the MAM, thus hampering the possibility to sustain ER-mitochondria interactions and their related function, through a loss of function mechanism.
